# Interview with Dr William Slikker Jr., Ph.D., Former Director, National Center for Toxicological Research, U.S. Food and Drug Administration (FDA), USA

**DOI:** 10.25122/jml-2022-1030

**Published:** 2022-10

**Authors:** Stefana-Andrada Dobran, Alexandra Gherman

**Affiliations:** 1 RoNeuro Institute for Neurological Research and Diagnostic, Cluj-Napoca, Romania; 2 Sociology Department, Babes-Bolyai University, Cluj-Napoca, Romania

**Interviewer: Ștefana-Andrada Dobran**

**Interviewee: Dr William Slikker Jr., PhD**.

Dr **William Slikker Jr**., Ph.D., served as Director of the National Center for Toxicological Research, U.S. Food and Drug Administration (FDA) until his retirement in March 2022.

This is an adapted Interview from the 15^th^ International Conference on Neuroprotective Agents (ICNA), that took place on September 11^th^–14^th^, 2022, Cluj-Napoca, Romania.

**Ș.A.D.:** Good morning! Dr Slikker, I have a few questions for you in the context of ICNA 2022.

**W.S.:** Good morning! Thank you!

**Ș.A.D.:** So, as the co-initiator of the International Conference on Neuroprotective Agents, would you be so kind as to share your first-hand opinion on this event?

**W.S.:** Well, thank you very much for the question! Well, [...] this being the 15^th^ conference, we do have some experience in this area, but we've been very excited about the opportunities here. Your team has taken very good care of us, and we have a very good arrangement of speakers, and the technical accommodations and support has been wonderful. But, I think the most important thing is to bring people together, and as this conference has done since the very beginning when Bruce Trembly and I…, Bruce Trembly passed away a few years ago, but he was trained as a neurosurgeon, I am trained as a pharmacologist/toxicologist, we decided, in 1991, that we needed to pull people together from various disciplines and really attack these issues of trying to protect the nervous systems. And so, to do that, we enlisted not only individuals in the neurosurgery and psychiatry area but also fundamental science areas, drug developers, regulators, and clinicians and clinicians in training. And so, from the very beginning, it's been important for us to have the opportunity to have people with various backgrounds work together to try to solve some of these issues and provide neuroprotection and also to be able to train individuals so that they can do it into the future. So, I think that the arrangements that have been made here for us have just been outstanding, and we certainly do really appreciate everything you have done to make this a really fantastic meeting. So, thank you!

**Ș.A.D.:** Thank you so much! We couldn't have done it without your input. So, my next question would be, considering your background, how would you describe the impact of neuroprotection on neurotoxicity in the context of the research presented at ICNA 2022?

**W.S.:** Well, I think that what we are learning here – and we are just at the end of the session on the influence of space travel on the nervous system, you can see the diversity of that compared to some of the other topics yesterday that dealt with more traditional neural insults, either due to drug exposures, anaesthesia, that sort of thing. So, it really is a diverse approach to understanding the impact on the nervous system, and I think that that sort of integrated approach is very positive. We're just learning new things today, you know, about intracranial pressure and its influence on the function of the nervous system and how you can monitor that to make sure that you have normal pressure within the brain. We're learning more about the imaging of the brain and doing quantitative assessments of brain function, as well as anatomy, so those things are overall useful. So, the development of really precise biomarkers is really key, and we're seeing a lot of that work today and also yesterday when we were looking at animal model work, trying to understand what biomarkers would be most effective and gaging how to protect the nervous system in humans, which is here [at the conference] the ultimate goal.

**Ș.A.D.:** Yes, thank you so much! And I have one last question for you regarding the pandemic since it's an ongoing subject. So, as the world enters the third year of the COVID-19 pandemic, doctors face more and more complex neurological cases. Considering your experience in this, what are the most relevant issues to be addressed in the near future?

**W.S.:** Yes, that's really a very important question and, you know, even though, as you said, we're entering the third year of individuals experiencing COVID-19, our understanding of the influence on the nervous system is still not really where [it is] needed to be. We are learning certain things, but unfortunately, some of those have to deal with [...] "brain fog" and that "brain fog" indicates that your nervous system does not function as it used to before exposure to COVID-19, before that infection. And it happens apparently in somewhere [between] 20% to 30% of individuals that have COVID-19 infection. It does tend to recover over time, but some people do not recover fully, so it's not anything that is trivial. We do not understand it very well at all at this point in time, so we're really working very hard to do that. I would say, from my perspective, as a human who is interested in the protection of the nervous system, that we oftentimes turn to animal models to try to understand [...] what is at the foundation of this problem – "brain fog" following COVID infection. And, you know, the animal models just have not been developed yet to really address this. So, I think that's one area that really needs to be strengthened so that we can understand more fundamentally what's going on. There seems to be [...] perhaps some immune functions involved in this, and also the shape and size of the brain. Many things seem to be going on – we don't understand them all, and we really need more fundamental work in that area. One sort of "bright spot", since we do have a lot of interest in developmental neurotoxicity, is a recent publication by Dr Sonja Rasmussen, who was at the University of Florida, now at Johns Hopkins, before that she was a long-time member of the Center for Disease Control in the US, CDC. Her study, along with other investigators, seems to indicate that there probably was not a birth defect associated with COVID-19 infection in pregnant moms. And this is a very positive outcome because so many things, of course, [...] can adversely affect development. But as far as her recent data – and it's new, and it's also limited – because there hasn't been that length of time, but the human data says, that probably it isn't a syndrome of developmental toxicity associated with COVID exposure with pregnant moms, as we know it right now. So, that was very positive to learn; there will be follow-up studies, I'm sure, on that, but I think that you know, all in all, that's really good news. So, we have a long way to go in understanding the influence of COVID on the nervous system, and it will take a combination of efforts, both animal model studies, and human studies, to really understand that and try to understand if there are any treatments that are possible.

**Ș.A.D.:** Alright, thank you so much for your interview and for your whole input for this conference!

**W.S.:** Well, thank you very much, and I really appreciate the efforts of the entire team working on this together. It's been a real delight, and I think it has influenced the entire operation's positive outcome. So, thank you!

**Ș.A.D.:** Thank you so much!

